# LOCAL ADAPTATIONS OF HIH: VARIATIONS IN STRUCTURE AND IMPLEMENTATION OF HIH MODEL ACROSS SIX VA PROGRAMS

**DOI:** 10.1093/geroni/igad104.0538

**Published:** 2023-12-21

**Authors:** Akanksha Samant, Sungjae Park, Emily Franzosa, Jennifer Sullivan, Porooshat Dadgostar, Derek Darcy, William Hung

**Affiliations:** James J. Peters VA Medical Center, Bronx, New York, United States; Icahn School of Medicine At Mount Sinai, New York City, New York, United States; Icahn School of Medicine at Mount Sinai, New York City, New York, United States; Center of Innovation in Long Term Services and Supports (LTSS COIN), Providence, Rhode Island, United States; University of Rochester Medical Center, Rochester, New York, United States; Canandaigua VA Medical center, Canandaigua, New York, United States; Icahn School of Medicine at Mount Sinai, New York City, New York, United States

## Abstract

A number of Department of Veterans Affairs (VA) medical centers have adopted the Hospital-In-Home (HIH) model of care to provide at home services to Veterans requiring acute-level hospital care. We aimed to identify similarities and variations in structure and implementation of the HIH model across VHA sites. Of 11 programs, six (55%) used a standardized presentation template to present their specific program characteristics, outcomes and goals at monthly national program meetings. Presentations were transcribed and abstracted into themes by two reviewers. Programs varied in their staffing, services provided, referral sources and clinical outcome metrics. While one site provided in person visits, 3 reported program adaptations such as virtual follow-up visits for COVID patients. Review of clinical outcome metrics also revealed substantial site to site variations including 30-day readmission rates, cost avoidance, bed days or emergency department visits. All sites offered complementary (from hospital) and substitutive (from other than hospital) HIH stays. Substitutive stays varied across referral sources admitting patients from emergency departments, outpatient clinics, home based primary care and inpatient settings. Additionally, variations in catchment area (driving distance), and staffing in the parent stations highlighted differing contexts of HIH programs. We hypothesize that variations among sites may exist due to factors such as the setting and unique needs of the population and facility. An in-depth understanding of the contextual factors that influence implementation can be achieved through interviews with key staff from each site. Future work will examine if such variations are related to program outcomes.

